# The maternal–fetal communicatome: a scoping review and network physiology perspective on a unified, adaptive, and multiscale interaction network

**DOI:** 10.3389/fnetp.2026.1864981

**Published:** 2026-09-07

**Authors:** Igor V. Lakhno

**Affiliations:** Department of Obstetrics and Gynecology, Kharkiv National Medical University, Kharkiv, Ukraine

**Keywords:** Barker hypothesis, fetal neurodevelopment, heart rate variability coupling, immune tolerance, maternal–fetal attachment, microchimerism, network physiology, placenta

## Abstract

The relationship between the mother and fetus is increasingly recognized not as a simple interaction between two separate entities but as a unified, adaptive, and multiscale physiological network. This scoping review synthesizes current research across psychological, physiological, immunological, genetic, and cellular domains to map the “communicatome” that orchestrates pregnancy and programs lifelong health. Centrally, we examine the coupling of maternal and fetal heart rate variability (HRV), a paradigmatic manifestation of network physiology in which maternal autonomic oscillations are mirrored in umbilical venous and ductus venosus blood flow to modulate fetal neurodevelopment. We conclude that the decoupling of this physiological network—detectable through integrated biosignal analysis—serves as a critical early-warning marker for pregnancy pathologies, including pre-eclampsia (PE), fetal growth restriction (FGR), and preterm birth (PTB). Furthermore, the review highlights the active nature of fetal–maternal interactions, in which fetal trophoblasts “educate” the maternal immune system to ensure semi-allogeneic tolerance, and microchimeric cells establish a biological legacy that persists for decades postpartum. Psychologically, prenatal attachment is shown to be a transgenerational process in which the quality of current relationship care mediates the transmission of ancestral parenting patterns. By framing these interactions through the Developmental Origins of Health and Disease, we conclude that the decoupling of this physiological network—detectable through integrated biosignal analysis—serves as a critical early-warning marker for pregnancy pathologies and neurodevelopmental vulnerabilities.

## Introduction

The relationship between a mother and her fetus is the most intimate human interaction, functioning not as two separate entities but as a unified, adaptive, and multiscale physiological network. This complex “communicatome” involves dynamic, bidirectional signaling at molecular, cellular, systemic levels that orchestrate pregnancy, maternal adaptation, and fetal maturation. Central to this dialogue is the placenta, which serves as the master regulator and primary interface, secreting hormones and extracellular vesicles (EVs) while acting as a sophisticated sensor for maternal nutritional and stress signals (Kareus et al., 2026; Kramer et al., 2023).

Maternal–fetal cross-talk encompasses dynamic, bidirectional signaling at molecular, cellular, tissue, and systemic levels that coordinates pregnancy, fetal development, and maternal adaptations. The human placenta is a unique organ that reacts sensitively to any pathological state within the unified mother–placenta–fetus system. It possesses compensatory capabilities and adaptive processes that “smooth out” deviations within the network. Morphological indicators of this connection include the state of the spiral arterioles of the endometrium and the syncytiotrophoblast. Disruptions in these connections, such as trophoblast failure to remodel maternal spiral arteries into high-capacitance vessels, lead to placental ischemia and oxidative stress—primary pathogenetic drivers of pre-eclampsia (PE). Critical mediators include placental-derived allopregnanolone (ALLO) and a diverse array of extracellular vesicles (EVs) that carry miRNA cargo, orchestrating a systemic reorganization of maternal and fetal networks (Opichka et al., 2025). The placenta serves as the primary interface, secreting hormones [e.g., human chorionic gonadotrophin (hCG), estrogens, and progesterone], growth factors [e.g., placental growth factor (PlGF)], anti-angiogenic factors [e.g., soluble forms like tyrosine 1 (sFLT1)], cytokines, and EVs carrying miRNAs and other cargo into both maternal and fetal circulations. This interface does not merely facilitate exchange; it actively modulates maternal physiology to support fetal metabolic demands while simultaneously guiding fetal immune tolerance and neural programming. These signals drive maternal cardiometabolic adaptations (e.g., increased cardiac output and insulin resistance) while modulating fetal organogenesis, immune tolerance, and metabolic programming. Additional layers include immune cross-talk at the feto-maternal interface [e.g., between trophoblasts, decidual natural killer (dNK) cells, and macrophages], microbial influences, and direct tissue interactions (e.g., fetal membranes and myometrium promoting pro-inflammatory shifts at parturition). Disruptions in this cross-talk link to complications such as PE, preterm birth (PTB), fetal growth restriction (FGR), and long-term offspring cardiometabolic risk via *in utero* programming (Sferruzzi-Perri and Camm, 2016).

Recent single-cell and multi-omics studies highlight the complexity: for instance, labor involves orchestrated maternal–fetal cellular dialogues in the placenta, while m^6^A RNA modifications act as sensitive regulators of placentation and fetal responses to maternal stressors. These processes operate across scales—from epigenetic and post-transcriptional modifications in individual cells to organ-level synchronization (e.g., maternal cardiovascular–fetal heart rate coupling) and organismal integration (mother–placenta–fetus as a unified triad) (Candia-Rivera and Chavez, 2025).

Within the emerging framework of network physiology, the mother–placenta–fetus unit is viewed as a dynamic triad in which subsystems—including cardiovascular, autonomic, endocrine, and immune systems—are coupled through nonlinear feedback loops (Ivanov et al., 2016). A paradigmatic manifestation of this integration is the coupling of maternal and fetal heart rate variability (HRV), which reflects synchronized autonomic nervous system (ANS) activity and provides a critical non-invasive window into fetal neurological maturation. These autonomic rhythms, which specifically refer to maternal autonomic rhythms (e.g., respiratory sinus arrhythmia at 0.5 Hz), are often “mirrored” in the hemodynamics of the umbilical vein, which conducts maternal oscillatory processes, such as respiratory sinus arrhythmia (RSA), across the placental barrier (Lakhno, 2016).

The integrity of this cross-talk is essential for healthy neurodevelopment as the intrauterine environment serves to program the physiological and metabolic set points for the offspring’s entire life course. This paradigm, known as the Developmental Origins of Health and Disease (DOHaD) or the “Barker Hypothesis,” posits that disruptions in these network couplings can predispose the individual to lifelong cardiometabolic, endocrine, and neuropsychiatric vulnerabilities (Faa et al., 2024). Furthermore, this relationship establishes a long-term biological legacy through the bidirectional exchange of microchimeric cells and the intergenerational transmission of psychological attachment patterns.

This scoping review synthesizes current research on these multidimensional interactions, emphasizing how the synchronization of maternal and fetal biosignals drives neurodevelopment and how the decoupling of this physiological network serves as an early-warning marker of pregnancy pathologies.

## Methods

To ensure methodological transparency and minimize bias, the search strategy and eligibility boundaries of this scoping review were guided by the SPIDER (Sample, Phenomenon of Interest, Design, Evaluation, Research type) framework (Table 1). This framework is specifically optimized for scoping reviews synthesizing complex, multi-system, and mixed-method biological phenomena. Following the identification of initial records, screening was performed based on these SPIDER boundaries, focusing strictly on bidirectional maternal–fetal interactions rather than isolated maternal or fetal clinical parameters. A total of 38 sources met all eligibility criteria and were selected for qualitative meta-synthesis.

**TABLE 1:** SPIDER search tool for literature eligibility.

SPIDER element	Scoping review eligibility criteria	Representative search keywords
Sample (S)	Pregnant human mothers, fetuses, and the placental interface	Pregnant mother, fetus, placenta, and maternal–fetal triad
Phenomenon of interest (PI)	Multiscale, bidirectional communication, autonomic/biophysical coupling, and molecular signaling	Communicatome, HRV coupling, extracellular vesicles, microchimerism, mTOR, and OGT
Design (D)	Scoping review methodology, thematic qualitative synthesis, and multiscale network mapping [reviewer 6]	Scoping review, systematic review, and meta-synthesis
Evaluation (E)	Gestational health, fetal neurodevelopment, network decoupling, and GOS/PTB outcomes	Fetal programming, DOHaD, preeclampsia, fetal growth restriction, preterm birth, and fABAS
Research type (R)	Qualitative, quantitative, mixed-methods, clinical cohorts, and preclinical/rodent mechanistic models [reviewer 6]	Empirical study, cohort, spatial transcriptomics, sand ingle-cell RNA-seq

**FIGURE 1:**
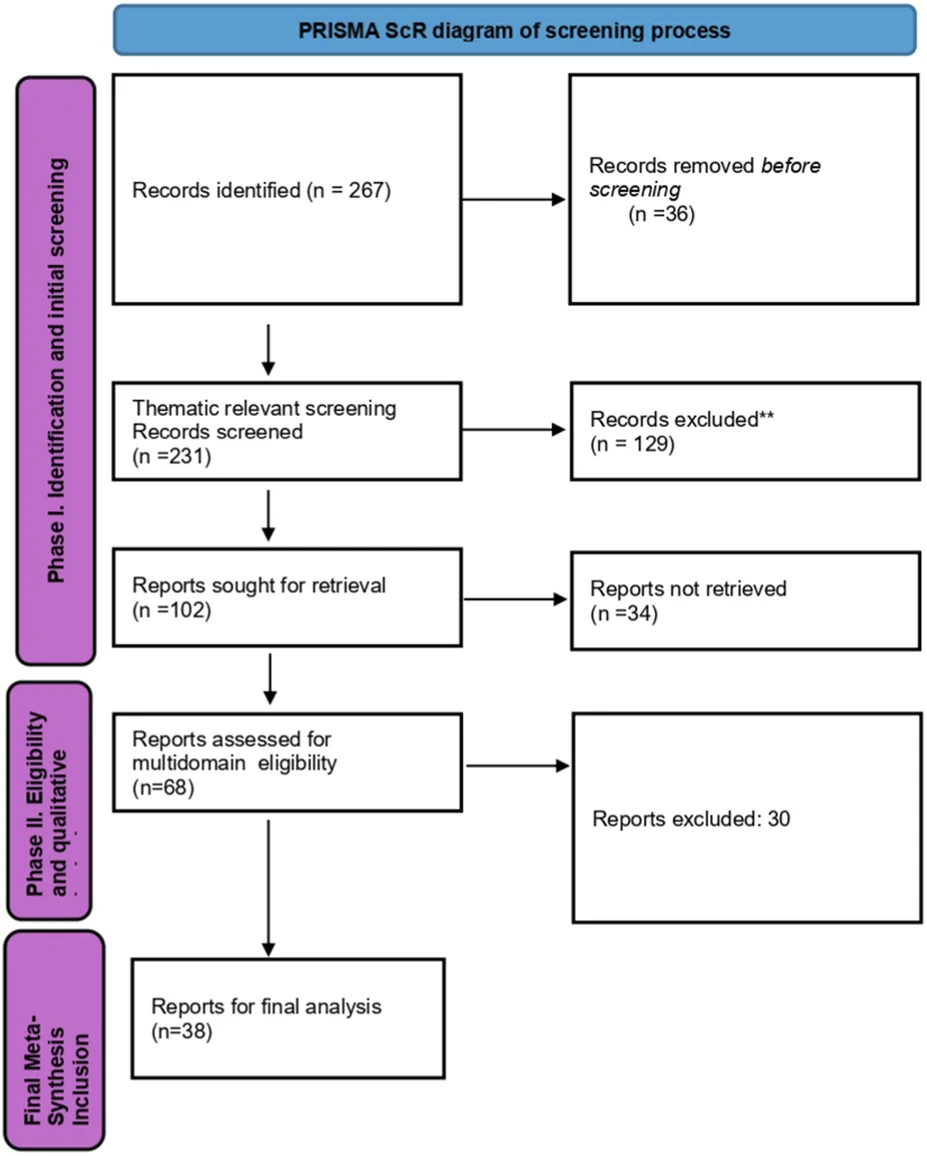
Stages of paper selection included in the study.

Flow diagram for maternal–fetal relationship review (see Figure 1).

1. Identification and initial screening phase. For this scoping review, a literature search was performed across PubMed, Scopus, and Web of Science (1996–2026) using the following search string: (‘maternal–fetal interaction’ OR ‘communicatome’) AND (‘Network Physiology’ OR ‘HRV coupling’ OR ‘fetal programming’). Records (n = 267) were assessed for their contribution to the bidirectional “communicatome.” Following PRISMA-ScR, all records were screened for thematic relevance (n = 231). The primary sources were evaluated for their contribution to the bidirectional “communicatome” between mother and fetus. Then, 163 records were excluded from the review. Records excluded during screening were removed due to a lack of thematic relevance to bidirectional networks or a focus on isolated clinical outcomes.
2. Eligibility phase. Full-text reports were assessed for multidomain eligibility (n = 68). Eligibility required studies to characterize interactions across at least two physiological subsystems or to characterize multiscale dynamics. The selected 68 sources were detected as each providing unique data for one of the five interaction domains (psychological, physiological, immunological, genetic, or cellular/microbial). The consolidation allowed us to ensure a consistent database for network physiology and fetal and maternal coupling analysis. Criteria: they must provide empirical data or theoretical frameworks regarding cross-talk, fetal programming, or intergenerational attachment. A total of 30 reports were excluded (n = 30). All selected manuscripts provided documents that met the multidisciplinary criteria for the integrated network analysis.
3. Inclusion phase. Studies included in the final meta-synthesis are presented in Table 2. Synthesis outcome: the integration of these 38 sources defines the mother–placenta–fetus triad as a unified, adaptive, and multiscale physiological network. Clinical integration: Data regarding the fetal Autonomic Brain Age Score (fABAS) and umbilical mirror were cross-referenced with DOHaD principles from the broader clinical set.

Level 1: Systematic input parameters (sample and phenomenon of interest)

**TABLE 2:** All included studies by domain.

Domain	Number of sources	Key mechanisms extracted
Psychological	3	Prenatal attachment predictors; intergenerational mediation
Physiological/metabolic	21	HRV coupling; umbilical vein spectral “mirroring”
Immunological	2	Trophoblast education of DICs; Treg essentiality
Genetic	4	Conflict theory; genomic imprinting (IGF-2/IGF-2R)
Cellular/microbial	8	Microchimerism; EV signaling; gut–brain axis (GBA)

Meta-synthesis. Thematic flow chart: SPIDER-aligned meta-synthesis extraction process.

(S)—Sample (target triad): Pregnant human mothers, fetuses, and the maternal–fetal–placental interface.

(PI)—Phenomenon of interest (the communicatome): Multiscale, bidirectional biological communication, autonomic cardiac coupling, and cellular/molecular signaling networks.

Level 2: Methodological filters (design and research type)

(D)—Design (synthesis protocol): Scoping review methodology mapping multidisciplinary evidence under a unified PRISMA-ScR framework to eliminate selection and reporting bias.

®—Research type (evidence base): Integration of human clinical cohorts (such as simultaneous NI-fECG and Doppler ultrasound databases) alongside preclinical/rodent mechanistic models.

Level 3: Qualitative data extraction (evaluation domains)

(E)—Evaluation (the five domains of interaction):

- Psychological domain (Andrek): Predictors of prenatal attachment (gestational age, fetal movement, and intendedness) and intergenerational mediation through current relationship care.
- Physiological and metabolic domain (Lakhno/Kramer): Beat-to-beat HRV/RSA coupling, umbilical venous spectral “mirroring” (0.45–0.55 Hz peak), and placental nutrient-sensing via the mTOR pathway.
- Immunological domain (Zhang): Active fetal trophoblast-driven “education” of maternal dNK cells and macrophages into tolerant, anti-inflammatory M2 phenotypes.
- Genetic domain (Haig): Evolutionary conflict theory and the parent-of-origin genomic imprinting tug-of-war (IGF-2 growth promotion vs. IGF-2R growth restriction).
- Cellular and microbial domain (Graf/Sajdel-Sulkowska): Decades-long persistent microchimerism, EV cargo traffic (eNOS, apoE, and nucleic acids), and maternal gut microbiota programming of the fetal gut–brain axis (GBA).
Level 4: Integrated meta-synthesis output (The Unified Network)
  - Horizontal integration: Bidirectional synchronization of independent maternal and fetal subsystems (cardiovascular, endocrine, immune, and neural).
  - Vertical integration: Propagation of sub-cellular, epigenetic changes (e.g., stress-regulated placental OGT and chromatin histone remodeling H3K27me3) to organismal-level adaptations.
  - Pathological decoupling: The failure of adaptive network communication acts as the shared pathogenetic signature of the great obstetrical syndromes (PE and FGR) and PTB.
  - Core conclusion: The mother, placenta, and fetus function as a unified, adaptive, and multiscale physiological network that programs lifelong health trajectories under the DOHaD paradigm.

## The review of the key findings

1. Key findings extracted from the psychological domain (prenatal attachment):
  - Prenatal attachment predictors: Linear regression models identify that gestational age at the point of assessment significantly predicts the intensity of maternal–fetal attachment, with bonding strength increasing as the mother perceives fetal movements. Maternal–fetal bonding is significantly predicted by the mother’s intendedness and happiness regarding the pregnancy; linear regression models identify gestational age, perception of fetal movements, and willingness to breastfeed as significant predictors among mothers (Jespersen et al., 2024).
  - Attachment theory evolution: Contemporary updates to attachment theory emphasize the long-term impact of prenatal and early-life separation; paternal–fetal attachment is significantly influenced by parity (first-time fathers show higher scores) and current relationship care; childhood experiences of care do not directly predict attachment but are mediated through the quality of care in the current relationship with the partner (Behrens et al., 2025).
2. Physiological and metabolic domain (network physiology). This domain defines the mother, placenta, and fetus as a unified triad characterized by multiscale autonomic and metabolic interactions. Network physiology framework: The mother and fetus are viewed as a dynamic network of interconnected systems where coordinated interactions produce health or pathology (Lin et al., 2020). The following key findings were extracted:
  - Quantifying network interactions: We quantify these dialogues as network interactions—nonlinear, time-varying couplings—using analytical tools such as partial directed coherence (PDC) to map the shift from fetal-to-maternal dominance to a stronger maternal-to-fetal drive (Candia-Rivera and Chavez, 2025; Ivanov et al., 2016).
  - Autonomic coupling: There is strong evidence for bidirectional interactions and synchronization between maternal and fetal heart rates, influenced by factors such as maternal respiration and fetal movement (Samjeed et al., 2024a); maternal and fetal heart rates exhibit time-varying couplings; the umbilical vein acts as a “mirror” of maternal autonomic oscillations (e.g., respiratory sinus arrhythmia at 0.5 Hz) (Lakhno, 2017).
  - Autonomic brain age and maturation: Fetal HRV metrics and behavioral states are used to estimate the “autonomic age” and maturation of the fetal central nervous system (Samjeed et al., 2024b; Hoyer et al., 2017).
  - Metabolic sensing: The placenta uses the mTOR signaling pathway to sense nutrients, prioritizing maternal supply over fetal demand to ensure mutual survival; the placenta acts as a master programmer of fetal metabolism and development, sensing maternal nutritional signals to set the lifelong health trajectory of the offspring (Kramer et al., 2023).
  - Fetal programming (the Barker hypothesis): Suboptimal intrauterine environments predispose the individual to chronic adult diseases such as hypertension and diabetes (Kwon and Kim, 2017).
  - Pathological decoupling in PE/FGR: Complications such as PE and FGR are associated with a breakdown in the circulation coupling between mother and neonate (Piani et al., 2026).
  - Stress regulation: The enzyme O-GlcNAc transferase (OGT) serves as a placental biomarker for maternal stress, regulating epigenetic programming in a sex-specific manner (Gao et al., 2026).
3. Immunological domain (maternal–fetal tolerance). The following key findings were extracted:
  - The immunological barrier: The maternal–fetal interface functions as a specialized barrier whose structure and regulation are vital for preventing the rejection of the semi-allogeneic fetus (Kareus et al., 2026).
  - Active education: Fetal trophoblasts actively “educate” maternal dNK cells and macrophages to adopt a tolerant, anti-inflammatory phenotype (M2) (Zhang et al., 2023).
  - Treg essentiality: Regulatory T cells (Tregs) are critical; their absence leads to uniform rejection of the semi-allogeneic fetus (Kareus et al., 2026).
  - Microbial role: Commensal uterine microbiota interacts with trophoblasts to maintain immune homeostasis and protect against viral infections (Barrientos et al., 2024).
4. Genetic domain (conflict theory). The following key findings were extracted:
  - The genetic node of the triad: The genetic pillar is characterized by the negotiation of resource allocation. Genomic imprinting—specifically the IGF-2/IGF-2R tug-of-war—acts as a vital node where paternal and maternal systems negotiate nutrient extraction. Similarly, the psychological domain is reframed as a transgenerational process in which the quality of current relationship care acts as a ‘funnel’ for transmitting ancestral parenting patterns through the network (Kareus et al., 2026);
  - Resource tug-of-war: Evolutionary theory posits a conflict where fetal genes (paternally derived) maximize nutrient extraction, while maternal genes limit it to preserve the mother’s health; maternal nutrition acts as a direct modulator of fetal gene expression, influencing susceptibility to metabolic disorders in adulthood (Andonotopo et al., 2025);
  - Genomic imprinting: The genetic pillar of the communicatome is characterized by the evolutionary dynamics of resource allocation between mother and fetus. Current research in this domain centers largely on genomic imprinting (e.g., IGF-2/IGF-2R) and genetic conflict theory, in which paternal and maternal genes negotiate nutrient extraction and conservation. However, it is important to note that the discussion within this domain remains relatively narrow compared with the physiological and autonomic pillars, focusing on a selected subset of imprinted regulatory pathways rather than a comprehensive genomic mapping of the triad.

Paternally expressed genes such as IGF-2 stimulate growth, whereas maternally expressed receptors such as IGF-2R act as growth inhibitors. Stress-induced RNA modification: maternal stress can induce placental disorders by modifying RNA m6A methylation (Hughes et al., 2019).

- Pathological outcomes: Conditions such as PE and gestational diabetes can be interpreted as manifestations of this ongoing genetic escalation (Andonotopo et al., 2025).

5. Cellular and microbial domain (chimerism and GBA). The following key findings were extracted:
  - Microchimerism: Fetal cells (FMc) and maternal cells (MMc) are exchanged and persist in the host’s tissues (brain, heart, and lungs) for decades, potentially aiding in tissue repair or influencing autoimmune health. Pregnancy involves a complex exchange of cellular and extravesicular chimerism that persists long after birth, influencing both maternal and fetal health (Graf et al., 2024).
  - EVs: Biological messages are “coded” into lipid-bound vesicles that pass through the placenta to modulate metabolic and immune systems; derived EVs can modify host dendritic cells to generate split immunological tolerance (Bracamonte-Baran et al., 2017).
  - GBA: Maternal gut microbial metabolites (e.g., short-chain fatty acids) cross the placenta to program the development of the fetal central nervous system and immune system; the maternal gut microbiome and nutrition during pregnancy are critical modulators of the fetal gut–brain axis and its long-term development (Sajdel-Sulkowska, 2023).
  - Chimerism shaping behavior: Maternal microchimerism has been shown in animal models to actively shape offspring neurodevelopment and behavior (Schepanski et al., 2022).

## How this work addresses interactions across systems/sub-systems, multiscale dynamics, and broader network-based physiological principles

The manuscript’s findings directly engage these dimensions by demonstrating coordinated interactions among maternal (cardiovascular, endocrine, and immune), placental, and fetal (metabolic, neural, and cardiovascular) subsystems. Rather than isolated effects, we observe emergent network behaviors: transient couplings (e.g., via placental EVs and cytokines) that synchronize maternal metabolic output with fetal growth trajectories, and feedback loops where fetal signals (e.g., through cord blood mediators) retroactively modulate maternal inflammatory networks. This aligns with multiscale dynamics, where molecular-scale changes (e.g., altered m^6^A or miRNA cargo) propagate to cellular invasion/migration at the implantation site, tissue-level remodeling (e.g., spiral artery transformation), and systemic adaptations (e.g., maternal left-ventricular hypertrophy or immune tolerance) (Kramer et al., 2023; Gao et al., 2026).

We quantify these as network interactions—nonlinear, time-varying couplings across physiological subsystems—using integrative analyses of biosignals and molecular profiles. Previous investigations by the authors using Ukrainian cohorts have demonstrated that under significant physiological stressors, such as those associated with wartime conditions, there is increased neighborhood connectivity in immune-mediator networks between maternal serum and fetal cord blood.

This suggests that the maternal–fetal triad functions as a redundant and cooperative system, utilizing these network interactions to enhance robustness during gestation (Lin et al., 2020; Hoyer et al., 2017).

## Placement of reported findings within the context of prior works on network physiology

These observations extend and operationalize the foundational framework of network physiology, as articulated by Ivanov et al. (2016), which views the human organism as a dynamic network of physiological systems whose coordinated, transient interactions across spatiotemporal scales generate health and distinct physiological states. Prior work in this field has explicitly identified maternal–fetal and neonatal care as key application domains, emphasizing how disruptions in inter-system couplings (e.g., cardio-respiratory or brain–heart) can cascade into pathology, while synchronized network dynamics enable adaptive states. The findings build on this framework by applying network principles specifically to pregnancy: we map maternal–fetal cross-talk as a multi-component network involving endocrine–immune–metabolic subsystems, with quantifiable couplings (e.g., phase synchronization or information transfer via placental signals) that reorganize across gestation (Lakhno, 2016; Lakhno, 2017). The review provides horizontal integration—this encompasses the transient, nonlinear synchronization of maternal cardiovascular, endocrine, and immune subsystems with fetal metabolic and neural counterparts—and vertical integration—this describes the propagation of signals across scales, where sub-cellular changes, such as m6A RNA modifications or miRNA cargo, drive tissue-level remodeling (e.g., spiral artery transformation) and organismal adaptations such as maternal left-ventricular hypertrophy (Candia-Rivera and Chavez, 2025; Lin et al., 2020). The outcomes of healthy and complicated pregnancies are presented in Table 3.

**TABLE 3:** Emergent network behaviors in the maternal—fetal dyad.

Feature	Normal gestation (synchronized)	Pathological states (decoupled)
Connectivity	High; coordinated metabolic and neural output	Low; “dissociation” between subsystems
Robustness	High; cooperation and compensation mechanisms	Low; vulnerability to stressors
Coupling	Strong bidirectional synchronization	Weakened; decoupled oscillatory dynamics
Signatures	Predictive of healthy maturation	Autonomic malprogramming; FGR/PE risk

## Discussion

### Integrative synthesis: a logic model of the maternal–fetal communicatome

**FIGURE 2:**
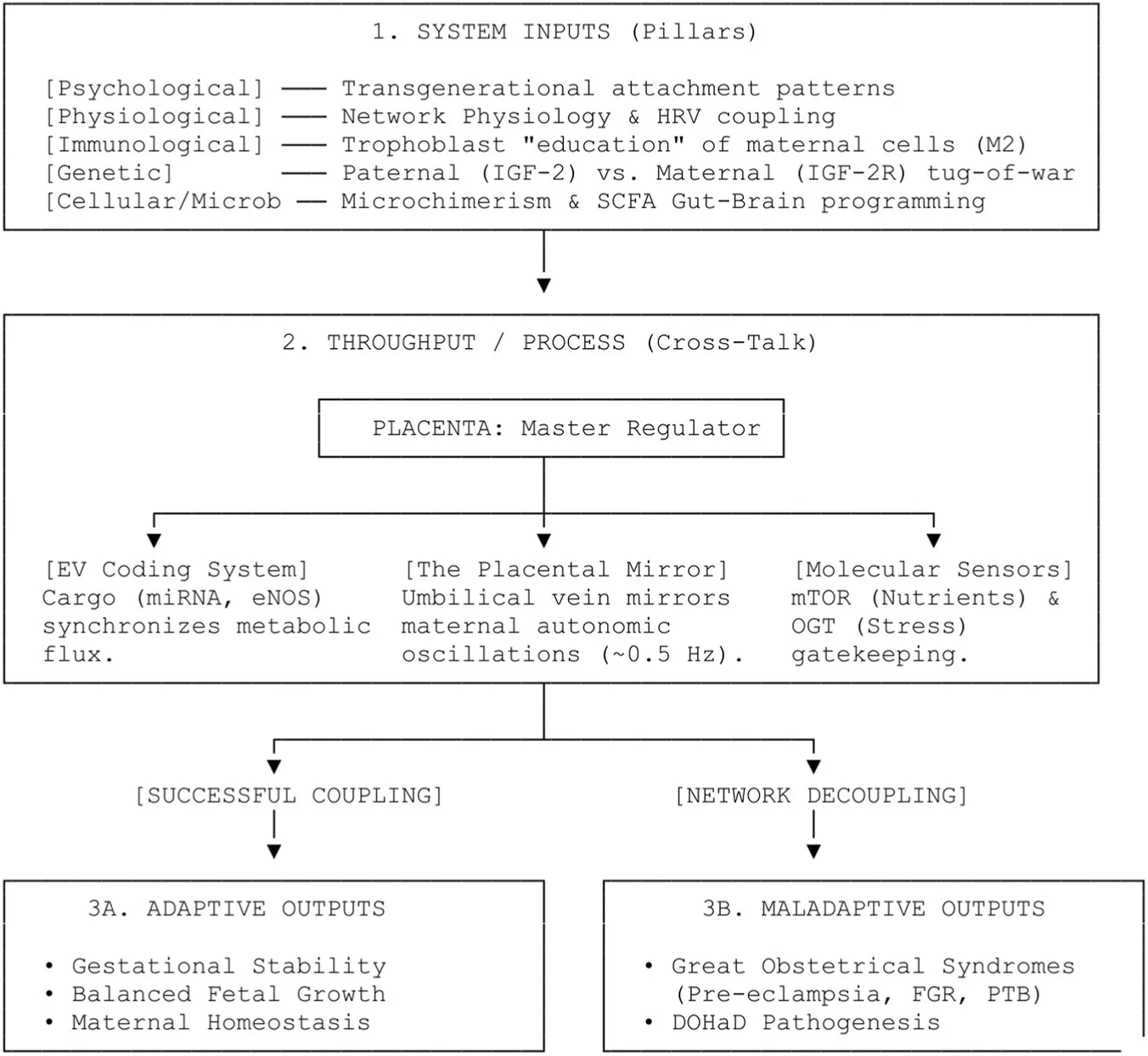
Logic model of the maternal–fetal communicatome.

Rather than viewing gestational pathologies as isolated organ failures, the synthesis of the 38 included studies allows us to construct an integrative logic model of the maternal–fetal communicatome (Figure 2). This model conceptualizes the mother–placenta–fetus triad as a system of coupled oscillators and molecular networks.

- System inputs (the five pillars): Bidirectional signaling is initiated across psychological, physiological, immunological, genetic, and cellular/microbial domains.
- Throughput (conduction and coupling mechanisms): The placenta functions as the master conductor, utilizing the non-innervated umbilical vein to mirror maternal autonomic oscillations (respiratory sinus arrhythmia at 0.5 Hz), while molecular sensors (mTOR and OGT) gate nutrient and stress transport.
- System outputs (coupling vs. decoupling): Healthy coupling yields optimized neurological maturation (high fABAS) and homeostatic metabolic programming. Conversely, network decoupling—such as the failure of trophoblast spiral arteriole remodeling or the stress-induced release of pro-inflammatory extracellular vesicles (carrying TNF, IL-6, and IL-8)—propagates system-wide disruptions. This molecular and biophysical decoupling acts as a common pathogenetic pathway, triggering pre-eclampsia, fetal growth restriction, and preterm birth, ultimately setting lifelong susceptibility to adult-onset chronic diseases under the DOHaD paradigm.

#### Maternal–fetal HRV coupling

The coupling between maternal and fetal HRV serves as a core manifestation of this cross-talk, reflecting synchronized ANS activity. The implications of maternal and fetal HRV are central to understanding the mother–fetus relationship as a unified, adaptive physiological network (DiPietro et al., 2023; Lakhno, 2025).

Directionality shifts: Using analytical methods such as partial directed coherence (PDC) and bivariate phase-rectified signal averaging (BPRSA), research demonstrates that heart rate coupling is time-varying. Early in gestation, fetal-to-maternal influence is often dominant, but this shifts to a stronger maternal-to-fetal drive by mid-to-late pregnancy (Nichting et al., 2023).

#### Placental mirror

The placenta and umbilical veins act as conductors of these oscillatory signals. The umbilical vein has no innervation and reflects oscillatory processes within the system of “mother–placenta–fetus.” A combined assessment of maternal–fetal HRV coupling and umbilical venous Doppler spectral analysis (for fluctuation peaks) offers a non-invasive window into placental conductive function and fetal wellbeing. This complements traditional cardiotocography and arterial Doppler. Specifically, spectral analysis of umbilical venous blood flow reveals a characteristic frequency peak at approximately 0.5 Hz that strongly correlates with maternal RSA and HRV. The 0.5 Hz frequency peak in the umbilical vein corresponds to the maternal respiratory rate, specifically representing a frequency range of approximately 0.45–0.55 Hz (corresponding to maternal RSA at ∼30 breaths per minute during paced breathing). The placental mirror operates as a bidirectional conductor. The fetus generates episodic breathing movements at a rate of 30–60 breaths per minute (∼0.5 Hz). This intrinsic fetal rhythm drives a local respiratory sinus arrhythmia that reflects through the umbilical vein, modulating placental blood flow and anchoring maternal–fetal autonomic synchronization (Kramer et al., 2023; Lakhno, 2016; Hoyer et al., 2017). Fetal breathing changes intrathoracic pressure. This pressure shifts blood volume within the low-resistance umbilical circulation. The resulting rhythmic mechanical waves travel through the umbilical vein back to the placenta. This mechanical feedback loops back into the maternal–fetal interface. It can influence maternal heart rate variability and synchronize the autonomic nervous systems of both individual organisms (Lakhno, 2017). The 0.5 Hz peak is absent or significantly diminished under pathological conditions such as PE or FGR, indicating a breakdown in network connectivity. The relationship between maternal and fetal root mean square of successive differences (RMSSDs) was found in healthy pregnancies. Maternal parasympathetic regulation and RSA had a protective impact on the circulatory responses of the mother and fetus in PE. The increased maternal autonomic balance supported perfusion of the end organs and coupling with fetal hemodynamics in mild–moderate PE, while vagally mediated reactions were safe. In pathological states such as severe PE, the authors’ previously reported data indicate that a hypokinetic type of combined maternal–fetal hemodynamics is associated with a significant reduction in RSA. In these instances, the fetal cardiovascular system appears functionally dissociated from the maternal organism, a state confirmed by the absence of significant correlations between maternal and fetal HRV parameters, including total power (TP) and RMSSDs (Lakhno, 2016; Lakhno, 2017). This “network decoupling” represents a failure of the normal adaptive communication between mother and fetus. For this reason, the loss of fetal and maternal hemodynamic coupling could be considered a presumable pathogenetic mechanism of fetal distress in PE (Kramer et al., 2023; Hoyer et al., 2017; Hoyer et al., 2019).

Maternal modulation: Maternal states, including breathing patterns, physical activity, and emotional stress, propagate through these autonomic couplings to influence fetal cardiac rhythms. For example, paced maternal breathing can enhance this synchronization (Hoyer et al., 2017; Bailey et al., 2024).

#### Fetal heart rate variability and neurodevelopment

Fetal growth and fetal HRV are closely linked, with fetal HRV serving as a critical non-invasive window into the physiological integrity and neurological maturation of the fetal central nervous system and the integration of brainstem and cortical networks (Hoyer et al., 2019). In healthy pregnancies, fetal HRV metrics (such as SDNN and RMSSD) typically increase as gestation advances, reflecting the progressive development of the ANS and a shift from sympathetic dominance toward vagal control. Under pathological conditions such as fetal growth restriction (FGR), these HRV metrics are often markedly reduced. This reduction in variability is a primary indicator of autonomic dysfunction and delayed neurological maturation in growth-restricted fetuses. Furthermore, the relationship between fetal growth and HRV is defined by the synchronization or “coupling” of maternal and fetal autonomic rhythms. In normal pregnancies, the umbilical vein acts as a physiological “mirror,” conducting maternal autonomic oscillations across the placenta to the fetus. In cases of FGR or PE, this network coupling often breaks down, leading to a “dissociation” that contributes to fetal distress, altered nutrient delivery, and impaired development (Hoyer et al., 2017).

The umbilical vein has been conceptualized as a non-innervated “physiologic mirror” of the oscillatory processes within the maternal–fetal-placental triad. Spectral analysis of umbilical venous flow velocity waveforms reveals characteristic frequency peaks—most notably a 0.5 Hz peak—that correlate directly with maternal HRV and RSA, illustrating the placenta’s capacity to conduct maternal autonomic fluctuations to the fetal compartment (Lakhno, 2017). However, although the umbilical vein provides a unique window into these vertical autonomic couplings, the ductus venosus is recognized as a more sensitive marker of fetal clinical status. The ductus venosus offers higher resolution for detecting early fetal cardiac compromise and compensatory responses to placental insufficiency. Consequently, although umbilical venous spectral analysis is indispensable for mapping the “mirroring” of autonomic rhythms, the inclusion of ductus venosus indices is essential for a robust clinical evaluation of fetal status. By prioritizing the ductus venosus alongside maternal–fetal HRV coupling, clinicians can more accurately detect the early signatures of network decoupling that precede severe pathologies such as PE and FGR (Hoyer et al., 2019).

Indices of maturation: As gestation advances, fetal HRV metrics (such as SDNN and RMSSDs) typically increase, while the baseline heart rate decreases. This progression mirrors the shift from sympathetic dominance toward greater vagal (parasympathetic) control. The development of fetal heart rate reactivity in a non-stress test was known as the fetal neurological maturation stage (Hoyer et al., 2017).

Autonomic brain age: Metrics such as the fetal Autonomic Brain Age Score (fABAS) are used to quantify this maturation (Hoyer et al., 2017). Stronger and more organized maternal–fetal HRV coupling is predictive of healthy ANS development and improved postnatal cognitive, motor, and regulatory outcomes (Hoyer et al., 2019).

Pathological decoupling: In pregnancies complicated by high maternal stress, inflammation, or placental insufficiency, this coupling is often weakened or decoupled. Reduced fetal HRV and decoupled rhythms are associated with increased risks for later psychopathology, temperament dysregulation, and executive function deficits (DiPietro et al., 2023; Chen et al., 2025).

The lower fetal HRV and short-term variation (STV) are moderate-to-strong biomarkers of fetal distress and acidemia, particularly in growth-restricted pregnancies. Long-term outcomes: Higher fetal HRV and steeper developmental trajectories after 28 weeks are predictive of better mental, psychomotor, and regulatory outcomes in early childhood. Decoupling could be a marker of major obstetric syndromes. The decoupling of the maternal–fetal heart rate network serves as an early-warning marker for FGR and other pregnancy pathologies, detectable weeks before birth. Although fetal growth is a complex process involving nutritional and hormonal factors, its success depends on the stability of the maternal–placental–fetal physiological network, for which HRV and autonomic coupling are primary descriptors (Kap et al., 2016; Kapaya et al., 2020).

#### Fetal HRV metrics as growth indicators

Specific fetal HRV metrics are directly linked to growth status. Fetuses suffering from growth restriction typically exhibit a “pathological pattern” of autonomic function (Lakhno, 2017; Hoyer et al., 2017; Lakhno, 2025; Hoyer et al., 2019):

- Reduced variability: FGR fetuses show markedly lower overall variability, including lower SDNN and STV.
- Sympathetic overdrive: these fetuses often have an elevated stress index (SI) and higher amplitude of mode (AMo), signaling autonomic malfunction and delayed neurological maturation.
- Delayed maturation: using the fABAS, clinicians can detect delayed maturation in growth-restricted fetuses weeks before clinical symptoms appear.

#### Broader scientific context and long-term associations

Independent studies reinforce these findings through the lens of developmental origins of health and disease (DOHaD/Barker hypothesis) (Hoyer et al., 2017; Nichting et al., 2023; Hoyer et al., 2019):

- higher fetal HRV (especially after 28 weeks) and steeper developmental trajectories predict better mental and psychomotor outcomes at age 2 years, as well as improved toddler cognition, language, motor skills, and regulatory behaviors;
- lower fetal HRV is associated with an increased risk for later psychopathology (internalizing/externalizing symptoms), altered temperament, executive function deficits, and behavioral inhibition;
- fetal autonomic programming influences lifelong ANS regulation: intrauterine growth restriction or stress can “program” reduced vagal tone, elevating risks for adult cardiovascular disease, hypertension, metabolic syndrome, and neurobehavioral disorders;
- postnatal HRV in newborns (influenced by fetal tone) continues to reflect these early imprints, with changes in the first hours/days of life linked to birth stress resolution and overall wellbeing.

#### Integration with maternal–fetal HRV coupling and network physiology

In the context of our ongoing discussion, the following can be obtained:

- Maternal–fetal HRV coupling: the coupling between maternal and fetal HRV serves as a core manifestation of this cross-talk, reflecting synchronized autonomic nervous system activity. Within the network physiology framework, these biosignals represent the beat-to-beat synchronization of two independent cardiac systems (Candia-Rivera and Chavez, 2025; Lakhno, 2016).
- Fetal autonomic tone is modulated by maternal–fetal HRV coupling and placental conduction of hemodynamic fluctuations (e.g., maternal RSA at ∼0.5 Hz influencing umbilical venous flow) (Lakhno, 2016; Lakhno, 2017).
- Stronger, organized coupling supports healthy ANS maturation; decoupling (as in preeclampsia/FGR) impairs vagal tone and accelerates sympathetic dominance, programming vulnerability (Hoyer et al., 2017).
- This exemplifies network physiology: transient, multiscale interactions in the mother–placenta–fetus network shape emergent autonomic states that program long-term adaptive capacity or disease risk (Ivanov et al., 2016; Lin et al., 2020).

Clinical implications: Non-invasive monitoring of fetal autonomic tone (via NI-fECG, magnetocardiography, or coupled HRV analysis), alongside umbilical venous Doppler, offers predictive value for neurodevelopmental trajectories and adult health risks. Placental bed pathology in FGR deteriorated the propagation of fluctuations and disrupted maternal–fetal interaction. The increased power of the maternal sympathetic domain region was revealed as a compensatory reaction due to deteriorated utero-placental hemodynamics. Sympathetic overactivity modulated maternal autonomic response by reducing TP and suppressing parasympathetic regulation. The role of the umbilical vein as a physiological “mirror” is supported by findings that a characteristic 0.5 Hz frequency peak in umbilical blood flow velocity waveforms correlates directly with maternal RSA. This maternal origin of the oscillatory signal has been established through research by Lakhno and others, highlighting the placenta’s capacity to conduct maternal autonomic fluctuations to the fetal compartment. Specifically, spectral analysis reveals a frequency peak (0.45–0.55 Hz) that correlates directly with maternal RSA, establishing the maternal origin of these fetal hemodynamic fluctuations (Lakhno, 2016; Lakhno, 2017; Hoyer et al., 2017). The value of the amplitude of the 0.5 Hz peak could be considered a biophysical marker of FGR and PE. As PE is a result of chronic placental insufficiency, the decreased amplitude of the above-mentioned peaks in maternal and fetal autonomic tone could be considered an additional criterion for such gestational disease. Early detection of autonomic malfunction enables timely interventions to mitigate programming effects. Fetal autonomic tone is not only a real-time marker of neurological maturation but also a prognostic window into future health—supported by clinical data and broader fetal programming research. Preserved vagal tone and intact maternal–fetal synchronization promote resilience; disruptions signal heightened lifelong vulnerability (Lakhno, 2017; Hoyer et al., 2017; Hoyer et al., 2019).

A central tenet of this synthesis is that the integrity of the maternal–fetal “communicatome” is essential for sustaining a healthy pregnancy. Although PE and FGR are primary focuses, PTB is a critical consequence of network decoupling. The transition to early delivery is often mediated by placental stress (e.g., deficiency in IFN-β signaling), which triggers the release of EVs with pro-inflammatory cargo (TNF-α, IL-6, and IL-8). This inflammatory cargo acts as a “molecular timer,” prematurely activating parturition pathways within fetal membranes and the myometrium. Specifically, EVs serve as a primary vehicle for this stress-induced communication; under pathological conditions, the placenta secretes vesicles with pro-inflammatory cargo—including specific miRNAs and cytokines—that can trigger the molecular cascades leading to parturition. For example, a deficiency in placental type I interferon-β signaling has been linked to increased production of pro-inflammatory cytokines such as TNF-α and IL-8, which are associated with the development of PTB. These cytokines, along with specific stress-induced miRNAs, are packaged into EVs and released into both circulations. This inflammatory cargo acts as a molecular “timer,” prematurely activating the parturition pathways within the fetal membranes and maternal myometrium, ultimately overriding the homeostatic signals required to maintain gestation. By recognizing PTB as a direct result of network decoupling, this framework allows for a more comprehensive understanding of how disruptions in molecular and autonomic synchronizations lead to a spectrum of adverse gestational outcomes (Gao et al., 2026; Jacobsen et al., 2025; Jacobsen et al., 2023).

### Evidence-based data on fetal HRV and short- and long-term outcomes

A formal, large-scale meta-analysis exclusively on prenatal fetal HRV metrics—such as STV from computerized cardiotocography (cCTG), SDNN, RMSSD, or frequency-domain indices—and both short- and long-term outcomes does not currently exist in the published literature. High heterogeneity in fHRV measurement methods (e.g., cCTG STV vs. non-invasive fetal ECG/magnetocardiography), gestational age at assessment, outcome definitions, and study populations (low-risk vs. high-risk, including FGR/PE) has limited pooled analyses. Scoping reviews explicitly note that variability in reporting precludes meta-analysis of neurodevelopmental links. However, targeted systematic reviews/meta-analyses and key prospective studies provide robust evidence. A synthesis is provided in the following section, with emphasis on STV (the most standardized and clinically used fetal HRV proxy) and integration with findings on autonomic tone in high-risk pregnancies.

## Comparison of fetal HRV metrics across studies

Within the framework of network physiology, the following fetal HRV metrics should be interpreted as quantitative signatures of the maternal–fetal communicatome. Rather than isolated variables, metrics such as STV and the SI provide a comparative baseline for understanding “network decoupling” in pathological states. For example, a dramatic shift in SI (reaching 1,437+ units) signals a breakdown in the adaptive autonomic network, where the fetal system becomes functionally dissociated from maternal rhythms. Fetal HRV metrics vary by measurement method (e.g., computerized cardiotocography/cCTG for STV and non-invasive fetal ECG or magnetocardiography for time/frequency-domain indices), gestational age (GA), behavioral state, and clinical status (normal vs. high-risk such as FGR or PE). Key metrics include the following (Hoyer et al., 2017; Hoyer et al., 2019; Kapaya et al., 2020):

- STV (ms): Primarily from cCTG; reflects beat-to-beat changes (vagal influence);
- SDNN (ms): overall variability;
- RMSSD (ms): short-term, parasympathetic/vagal activity;
- pNN50 (%): parasympathetic marker;
- frequency-domain: TP (total power), VLF, LF (sympathetic/mixed), and HF (parasympathetic);
- others: SI, AMo, LTV (long-term variation), and AC/DC (acceleration/deceleration capacity).

## Normative trends with gestational age (healthy pregnancies)

Fetal HR baseline decreases, while overall variability increases with advancing GA, reflecting ANS maturation (sympathetic early → increasing vagal dominance later). The dynamics of the development of cardiac function and autonomic regulation is as follows (Hoyer et al., 2017; Kap et al., 2016; Kapaya et al., 2020):

- fetal HR baseline (bpm): ∼151 ± 16 (first trimester), ∼145 ± 6 (second), ∼125–140 (third); normative means decrease from ∼147 bpm (23–27 weeks) to ∼142 bpm (30–34 weeks) to ∼137–141 bpm (36–39 weeks);
- STV (cCTG): increases with GA; first percentile ∼3.2–4.6 ms overall. Typical values increase from lower levels pre-32 weeks;
- SDNN and RMSSD (fMCG/fECG): both increase with GA; SDNN shows a clearer increase in active states. RMSSD is more stable but increases post-28 weeks;
- Complexity (e.g., ApEn and MSE): increases, indicating maturing neural integration.

Fetal movements modulate metrics: breathing increases RMSSD/HF (parasympathetic); body movements increase SDNN/LF (sympathetic shifts). The non-stress test is integrated into the current program of fetal wellbeing assessment (Hoyer et al., 2017; Hoyer et al., 2019).

## Comparison: normal vs. pathological pregnancies (FGR and PE)

High-risk pregnancies show reduced overall variability, delayed maturation, sympathetic dominance (higher SI/AMo and LF/HF), and weakened maternal–fetal coupling (Lakhno, 2017; Hoyer et al., 2017).

As this review acknowledges, high heterogeneity in fHRV measurement methods and the current absence of a formal, large-scale meta-analysis preclude the establishment of universal normative standards for the fetal autonomic nervous system. Consequently, the specific values presented in this synthesis—such as an SI of ∼141 units, TP reaching ∼16,000+ ms^2^, and an AMo of ∼38% in control groups—are intended as illustrative ranges derived from individual research cohorts.

These data provide a comparative baseline for understanding “network decoupling” in pathological states. For example, in cohorts of severe preeclampsia or fetal growth restriction, these metrics shift dramatically (e.g., AMo increases to 61%–100%, while SI can reach 1,437+ units), signaling a breakdown in the adaptive autonomic network. Clinicians and researchers should weigh these specific quantitative claims as study-specific benchmarks rather than definitive population norms. A progressive decrease was found in time-domain measures (SDNN and RMSSD) and power with increasing severity, along with sympathetic overdrive (high SI/AMo) and loss of coupling with maternal HRV (Lakhno, 2017). Similar patterns were observed in fMCG studies: lower SDNN/RMSSD in IUGR (Hoyer et al., 2019). Typical patterns of fetal HRV variables are presented in Table 4.

**TABLE 4:** Typical patterns of HRV variables (simplified; consult originals for exact GA/context).

Metric	Normal trend (↑ GA)	Normal range (approx.)	Pathological (FGR/PE)	Discriminatory value
STV	Increases	4–9+ ms	Decreased	Moderate (acidemia)
SDNN	Increases	30–50+ ms	Strongly decreased	High
RMSSD	Increases	8–22+ ms	Decreased	High (vagal)
SI/AMo	Lower	Lower	Markedly increased	Very high
TP/HF	Increases	Higher	Decreased	High

Illustrative ranges of fetal HRV parameters in healthy and pathological pregnancies. These values represent findings from specific research cohorts (e.g., Lakhno et al., Hoyer et al.) and should not be interpreted as universal normative standards due to the high heterogeneity of measurement protocols in the present literature.

## Short- and long-term outcome links

- Short-term (acidemia, Apgar, and distress): Lower STV/SDNN/RMSSD predict risk (Kapaya meta: moderate sensitivity/specificity for acidemia) (Kap et al., 2016). SI/AMo is highly sensitive for distress (Hoyer et al., 2019).
- Long-term (neurodevelopment): Higher fHRV trajectories (post-28 weeks) predict better Bayley scores at 2 years; reduced variability links to programming risks (DOHaD) (Hoyer et al., 2017; DiPietro et al., 2023).

### Key differences and challenges across studies

- Methods: cCTG (STV-focused, clinical) vs. fECG/fMCG (detailed time/frequency/nonlinear) → direct comparison limited (Ivanov et al., 2016; Lakhno, 2017; Hoyer et al., 2017; Hoyer et al., 2019).
- GA and state: variability increases with GA; behavioral state affects values significantly (DiPietro et al., 2023).
- heterogeneity: high in meta-attempts due to protocols, risk profiles, and definitions (Kap et al., 2016; Kapaya et al., 2020);
- network view: reduced metrics reflect the decoupled mother–placenta–fetus network, impaired placental conduction of fluctuations (∼0.5 Hz maternal RSA), and delayed ANS maturation (Ivanov et al., 2016; Lakhno, 2017; Hoyer et al., 2017).

## Short-term outcomes (perinatal: acidemia, apgar scores, NICU admission, and distress)

Existing meta-analysis: systematic review and meta-analysis of seven studies (780 singleton pregnancies ≥24 weeks) evaluated antepartum STV (via Dawes–Redman cCTG) for predicting fetal acidemia at birth (umbilical artery pH < 7.00 or <7.20, depending on study) (Kap et al., 2016). Pooled diagnostic accuracy (random-effects bivariate model) exhibits the following values:

- sensitivity: 0.57 (95% CI: 0.45–0.68);
- specificity: 0.81 (95% CI: 0.69–0.89);
- positive likelihood ratio (+LR): 3.14 (95% CI: 2.13–4.63);
- Negative likelihood ratio (−LR): 0.58 (95% CI: 0.46–0.72).

FGR subgroup: slightly improved detection (sensitivity 0.63 [95% CI: 0.49–0.75]; −LR 0.50 [95% CI: 0.31–0.80]).

Interpretation: STV shows *moderate* accuracy as a stand-alone test. Low STV (<∼3.5–5.25 ms, study-specific thresholds) flags risk of acidemia but misses many cases (moderate sensitivity). High specificity and good −LR support its use to *rule out* acidemia when STV is normal. Heterogeneity arose from varying pH thresholds, GA, and risk profiles; quality was generally high (STARD/QUADAS).

Intrapartum context: STV performed poorly (ROC AUC 0.527, 95% CI: 0.377–0.678) for umbilical artery pH < 7.20. Labor events (e.g., decelerations) altered STV dynamically, reducing utility compared to antepartum use (Kapaya et al., 2020).

### Apgar scores and other short-term markers

- Individual studies link lower fHRV/STV or higher coefficient of variation (CV) of fetal HR to lower Apgar scores (1- and 5-min) and metabolic acidosis (Lakhno, 2025; Nichting et al., 2023; Hoyer et al., 2019; Kapaya et al., 2020);
- In high-risk cohorts with PE/FGR, reduced fetal autonomic tone (lower SDNN and higher sympathetic indices such as stress index/AMo) correlates with fetal distress, lower Apgar, and autonomic imbalance; umbilical venous hemodynamics “mirror” this decoupling.
- FHR category meta-analyses (including reduced variability as a category II/III feature) show graded risk: category III tracings markedly elevate Apgar <7 and pH < 7.00 odds.
Lower fHRV/STV is associated with increased risks of acidemia, low Apgar, and distress, particularly in FGR/PE (consistent with network physiology framing of maternal–fetal decoupling). Predictive value is moderate antepartum but limited intrapartum.

#### Long-term outcomes (neurodevelopment and DOHaD programming)

No dedicated meta-analysis exists; evidence comes from prospective cohorts and scoping reviews. Heterogeneity note: studies vary in fHRV metrics, follow-up duration (infancy to childhood), and confounders (GA, sex, and maternal factors). Male fetuses often show higher HRV; maternal metabolic health modulates associations. Key findings are as follows:

- Landmark longitudinal study (n≈∼700 fetuses). Higher fHRV (and steeper developmental trajectories) at/after 28 weeks strongly predicted better mental and psychomotor outcomes at 2 years (Bayley Scales). Lower fHRV is linked to poorer regulatory capacities and neurobehavioral profiles (DiPietro et al., 2023).
- Scoping reviews of neonatal HRV (postnatal proxy for fetal programming) in preterm/perinatal risk groups consistently show decreased HRV associated with worse short- and long-term neurodevelopmental outcomes (e.g., cognitive/motor delays and cerebral palsy risk). A 2026 meta-analysis of *post-NICU* HRV (27 studies, 1,890 participants) found modestly reduced HRV in perinatal risk groups (overall Cohen’s d = −0.24), strongest in congenital heart disease/genetic syndromes and growth restriction, attenuating with age (Hoyer et al., 2017; Hoyer et al., 2019).
- Fetal programming (DOHaD): reduced prenatal fHRV (via maternal stress, IUGR, and inflammation) is linked to later psychopathology risk, altered temperament, executive dysfunction, and cardiometabolic vulnerability. Lakhno’s studies frame this as autonomic malprogramming from disrupted maternal–fetal coupling (Faa et al., 2024; Hoyer et al., 2017).

#### Molecular and microbial scaffolding

Although HRV coupling reflects a systemic biosignal, it is supported by molecular dialogue. The “communicatome” is supported by underlying molecular and microbial dialogues that serve as the network’s foundational coding system. EVs function as sophisticated, bidirectional messengers carrying multicomponent cargo—including eNOS for vascular regulation and apoE for metabolic adaptation—that synchronize maternal metabolic output with fetal growth trajectories. This scaffolding provides the multiscale logic that enables organ-level synchronization. The placenta acts as a master regulator, utilizing mTOR signaling as a network node to prioritize maternal resource stability. EVs, known as lipid-membrane-bound particles, including exosomes and microvesicles, function as a sophisticated bidirectional “coding system” between the feto-placental unit and the mother (Kareus et al., 2026).

Functional implications: Placental EVs carry a diverse cargo of bioactive signaling lipids, proteins, and nucleic acids (miRNA, mRNA, and DNA) that synchronize maternal metabolic output with fetal growth trajectories. For instance, syncytiotrophoblast-derived EVs modulate maternal insulin sensitivity to regulate glucose homeostasis, while MMc-derived EVs in the fetal compartment promote “split tolerance” by cross-dressing fetal antigen-presenting cells with maternal MHC molecules (Bracamonte-Baran et al., 2017).

Within the maternal–fetal “communicatome,” EVs function as a sophisticated, bidirectional “coding system” that transcends simple signaling. Although early research primarily emphasized their microRNA (miRNA) cargo, it is now recognized that EVs carry a much broader repertoire of biological mediators, including bioactive signaling lipids, proteins, and various types of nucleic acids, such as messenger RNA (mRNA) and DNA. These lipid-membrane-bound particles are continuously secreted by fetal-derived trophoblasts and maternal immune cells into both circulations, with their concentration increasing significantly throughout gestation (Gao et al., 2026).

The functional implications of this diverse cargo are multiscale:

Proteins and lipids: EVs transport functional proteins, such as active endothelial nitric oxide synthase (eNOS), which promotes maternal vasodilation, and bioactive lipids such as apolipoprotein-E, which can reach the maternal liver to modulate lipid metabolism. It targets the maternal liver to modulate cholesterol synthesis and lipid metabolism, thereby adapting maternal resource output to fetal demand. In PE, the activity of EV-bound eNOS is significantly impaired, contributing to maternal hypertension and network decoupling.

Nucleic acid repertoire: Beyond miRNAs, the presence of mRNA and DNA within these vesicles allows for the potential transfer of complex genetic instructions and epigenetic modifiers between the mother, placenta, and fetus. EVs facilitate the transfer of DNA and mRNA, which can “cross-dress” fetal antigen-presenting cells with maternal MHC molecules. This process induces “split tolerance,” a unique state where the fetal immune system recognizes maternal antigens to prevent rejection while maintaining the immunological memory necessary for lifelong health.

Systemic integration: This multifaceted cargo enables EVs to act as endocrine and paracrine mediators that synchronize maternal metabolic output with fetal growth trajectories.

By recognizing that EVs contain a complex “macro-molecular containment” rather than just isolated RNA species, we can better understand how the placenta functions as a master regulator, utilizing these vesicles to maintain adaptive homeostasis or, in cases of network decoupling, to communicate placental stress signals that contribute to pathologies such as PE and PTB (Gao et al., 2026).

The “communicatome” represents the multifaceted biological mediators—cells, vesicles, and soluble factors—that code signals between mother and fetus. Although HRV coupling is a systemic biosignal, it is supported by molecular and microbial dialogues that program the fetal GBA. EVs: The communicatome includes four distinct categories: endosomal origin (50–150 nm), plasma membrane budding (200–1,000 nm), apoptotic bodies: products of programmed cell death, and syncytial nuclear aggregates—unique placental vesicles (>20 µm) containing multiple nuclei, secreted by the syncytiotrophoblast (Sajdel-Sulkowska, 2023; Jacobsen et al., 2023). The main facts in the field are as follows:

- Metabolic sensors: the placenta uses the mTOR signaling pathway to sense maternal nutrient availability, synchronizing fetal growth trajectories with available maternal resources (Barrientos et al., 2024; Sajdel-Sulkowska, 2023).
- Stress programming: in rodent models, the enzyme O-GlcNAc transferase (OGT) has been identified as a critical cellular mechanism that senses maternal energy levels and stress. Experimental data in mice demonstrate that maternal stress hormones can activate glucocorticoid receptors, leading to a reduction in placental OGT levels. This reduction is associated with the destabilization of the histone methyltransferase EZH2 and a subsequent decrease in the transcriptional repressive mark H3K27me3. Although higher OGT expression in female placentas appears to provide a degree of epigenetic resilience, preclinical studies in rodents suggest that male fetuses may be more susceptible to maternal stress because their baseline OGT levels are naturally lower. This has led to the hypothesis that male individuals may fall below a “threshold of vulnerability,” impacting their neurodevelopment and growth trajectories. Although sex-specific differences in OGT expression have been confirmed in human placental tissue, the causal link between these epigenetic shifts and specific neurodevelopmental disorders remains an area of active clinical investigation. Thus, the enzyme OGT in the placenta acts as a biomarker of maternal stress; its inhibition can reprogram fetal hypothalamic gene expression, potentially impacting neurocognitive development (Gao et al., 2026).
- Microbial influence: maternal gut microbial metabolites, such as short-chain fatty acids (SCFAs), cross the placenta to program the development of the fetal central nervous system and blood–brain barrier. Depletion of this microbiota during pregnancy has been linked to deficiencies in thalamocortical neurodevelopment (Andonotopo et al., 2025).
- Chimeric and extravesicular messengers: naturally acquired chimerism involves the bidirectional exchange of FMc and MMc. FMcs are vertically transferred to the mother during gestation and establish a long-term biological legacy by engrafting into various maternal tissues. The duration of this persistence is generally characterized in the literature as lasting for decades. Although specific longitudinal research—notably the foundational work of Bianchi et al. (1996)—has identified male fetal progenitor cells in maternal blood as long as 27 years postpartum, this figure represents the maximum observed persistence in a specific study rather than a universal clinical constant (Bianchi et al., 1996). Therefore, FMc should be viewed as persistent cellular residents that influence maternal health trajectories over several decades. This “microchiome” with MMc engrafts in the thymus, bone marrow, thyroid, and brain. EVs and microchimeric cells further facilitate this communication, carrying lipids, RNA, and proteins that modulate fetal organogenesis, immune tolerance, and metabolic programming (Jacobsen et al., 2025; Jacobsen et al., 2023).

#### Microchimerism and fetal neurodevelopment

The long-term impact of MMc on the offspring’s brain has been explored through preclinical murine models. For instance, studies in mice have demonstrated that MMc can actively shape neurodevelopment and behavior by suppressing microglia activation and reducing the elimination of presynaptic vesicles (Schepanski et al., 2022). These rodent-based findings suggest a protective role for maternal cells in cognitive and behavioral development; however, although MMc have been detected in various regions of the human maternal and fetal brain, the functional implications for human neurodevelopment remain largely inferred from these animal models.

#### Fetal growth and development are significantly influenced by maternal stress

This impact is mediated through complex physiological, molecular, and microbial pathways centered primarily on the placenta. Although the literature on Tregs and genomic imprinting often focuses on isolated biological pathways, this review layers a synthetic network physiology framework onto these findings. We propose that the commonality among these disparate domains is their contribution to a unified, multiscale triad. For instance, the mTOR signaling pathway functions not only as a nutrient sensor but also as a network node that prioritizes maternal resource stability over fetal demand to ensure mutual survival. Mechanistic insights into placental OGT signaling, primarily derived from rodent models, suggest that this enzyme acts as a critical link between maternal stress and fetal hypothalamic programming, although further clinical validation in humans is required (Kramer et al., 2023; Sajdel-Sulkowska, 2023).

1. The placental stress sensor—OGT. A key mechanism linking maternal stress to fetal outcomes is the enzyme OGT (Gao et al., 2026; Barrientos et al., 2024; Sajdel-Sulkowska, 2023; Liu et al., 2024):
  - Glucocorticoid influence: when a mother experiences stress, the activation of the glucocorticoid receptor (GR) by stress hormones results in reduced OGT levels in the placenta.
  - Nutrient transport: reduced OGT levels alter the composition and number of EVs secreted into maternal circulation and can negatively impact placental nutrient transport.
  - Sex-specific vulnerability: in animal models, chronic stress early in pregnancy significantly reduces OGT throughout the remaining gestation period. This effect is more profound in male fetuses as they naturally have lower baseline levels of OGT, potentially falling below a “threshold of vulnerability” that impacts their neurodevelopment and growth. Beyond transcriptomic regulation, research in animals suggests that placental OGT may also regulate the biogenesis and cargo of extracellular vesicles. Specifically, in mice, OGT appears to modify annexin A1, a protein vital for EV packaging and secretion (Schepanski et al., 2022). Observations in these rodent models indicate that maternal stress reduces the levels of O-GlycNAcylated annexin A1, which could dynamically alter the composition and number of EVs secreted into the maternal circulation. Although these mechanisms provide a plausible pathway for how maternal stress signals reach the fetus, human studies currently rely on correlational evidence regarding EV cargo and maternal glucose homeostasis.
2. Hormonal reprogramming (HPA axis). Maternal stress leads to dysregulation of the hypothalamic–pituitary–adrenal (HPA) axis. The possible mechanisms of stress-related growth disorders are associated with the following:
  - Cortisol transfer: increased levels of circulating maternal cortisol can cross the placental barrier (Bailey et al., 2024).
  - FGR: the fetus adapts to these changes by increasing the production of catabolic hormones such as glucocorticoids, which can slow the rate of fetal growth and lead to intrauterine growth restriction (Hoyer et al., 2017).
  - Long-term programming: exposure to high levels of stress-induced corticosteroids can produce permanent changes in fetal neural pathways, predisposing the offspring to metabolic and cardiovascular diseases in adulthood, a concept known as the “Barker Hypothesis” or DOHaD (Sferruzzi-Perri and Camm, 2016; Faa et al., 2024).
3. Microbial and autonomic disruptions. The impact of stress is presented in Table 5. Stress also affects the integrated “communicatome” between the mother and fetus through other systemic channels:
  - Gut–brain axis: maternal stress induces rapid and lasting alterations in the maternal gut microbiota. This dysbiosis alters the metabolites (such as short-chain fatty acids) that cross the placenta to support fetal growth and brain development (Sajdel-Sulkowska, 2023; DiPietro et al., 2023).
  - HRV coupling: Maternal emotional stress propagates through synchronized HRV couplings. Chronic stress can weaken this synchronization, leading to a “decoupling” of the maternal–fetal physiological network, which is often a marker for fetal distress and impaired maturation (Lakhno, 2017; Hoyer et al., 2017).

**TABLE 5:** Summary of the stress impact.

Stress mechanism	Physiological effect	Fetal outcome
Placental OGT	Reduced nutrient sensing and vesicle cargo	Sex-specific neurodevelopmental risk
Cortisol/HPA axis	Catabolic hormone dominance over growth factors	Fetal growth restriction (FGR)
Gut microbiota	Altered metabolite production and transfer	Impacted neurocognitive development
Autonomic coupling	Weakened HRV synchronization	Increased risk for psychopathology

The relationship between OGT and FGR is defined by OGT’s role as a master placental sensor that links maternal stress and nutrient availability to fetal developmental programming. OGT is an enzyme that catalyzes the post-translational modification of proteins, placing it at the “crossroads” of nutritional signaling and gene regulation (Sajdel-Sulkowska, 2023). The following mechanisms describe how OGT influences fetal growth and the risk of restriction:

1. The maternal stress–placental OGT axis: Maternal stress is a primary driver of reduced placental OGT levels. When a mother experiences stress, the resulting elevation in stress hormones activates the GR, which directly results in reduced OGT levels in the placenta. This reduction has several downstream effects on the fetal environment: reduced nutrient transport—decreased OGT can negatively impact the transport of essential nutrients across the placenta to the fetus, a hallmark of FGR; epigenetic reprogramming—OGT stabilizes the histone methyltransferase EZH2, which maintains transcriptional repressive marks (H3K27me3). Reduced OGT leads to a loss of these marks, making the placental transcriptome more reactive to environmental insults, which can disrupt stable growth trajectories.
2. Sex-specific vulnerability and FGR: OGT is located on the X chromosome and escapes X-inactivation in placental tissue, meaning that female (XX) placentas naturally express higher levels of OGT than male (XY) placentas. Threshold of vulnerability: because male fetuses start with lower baseline OGT, maternal stress can push their OGT levels below a critical “threshold of vulnerability.” Impact on male fetuses: This makes male fetuses significantly more susceptible to growth restriction and neurodevelopmental impairments when exposed to early-gestation maternal stress compared to female fetuses, who have a larger “buffer” of OGT.
3. Regulation of EVs: OGT acts as a dynamic regulator of EV secretion by modifying annexin A1, a protein essential for packaging and releasing these vesicles (Liu et al., 2024). Homeostatic signaling: OGT-driven EVs are released into maternal circulation to help regulate maternal glucose tolerance and metabolic health.
  Pathological decoupling: when OGT is inhibited by stress, the composition and number of these biological “messages” are altered, potentially contributing to metabolic dysregulation that further restricts fetal resource supply.
4. Link to neurocognitive growth: Although OGT’s impact on physical growth is significant, it is equally critical for the growth of fetal neural pathways. Inhibition of placental OGT has been shown to reprogram fetal hypothalamic gene expression, linking restricted intrauterine conditions to long-term neurocognitive and neuropsychiatric risks in the offspring. OGT serves as a “placental biomarker” for maternal stress. Its reduction disrupts the nutrient-sensing and transport mechanisms of the placenta, creating a suboptimal environment that leads to fetal growth restriction, particularly in male offspring.

### Several biochemical and therapeutic strategies for improving or supporting fetal growth

The possible therapeutic strategies for improving fetal growth in FGR are as follows (Kramer et al., 2023; Sferruzzi-Perri and Camm, 2016; Zhang et al., 2023):

- Adiponectin normalization: Normalizing maternal circulating adiponectin levels in obese models has been shown to prevent the overactivation of placental nutrient transport and restore healthy fetal growth trajectories.
- Growth factor delivery: Experimental treatments have used viral vectors or nanoparticles to deliver IGF-1 or IGF-2 constructs directly to the placenta, which has successfully restored normal fetal weights in animal models of growth restriction.
- VEGF gene therapy: In cases of poor blood flow leading to restriction, adenovirus-mediated delivery of VEGF to the uterine artery has been shown to increase fetal growth velocity.
- Metabolic sensing: Fetal growth is also dependent on placental nutrient sensors such as mTOR and OGT, which adapt the supply of resources to the fetus based on maternal stress and nutritional availability.

In summary, the data focus on growth factor gene therapy, metabolic sensing pathways, and maternal microbial management as the primary prospects for improving fetal growth outcomes.

## Network interactions in the mother–placenta–fetus triad shape fetal neurodevelopment and maternal neuroplasticity

In the framework of network physiology, the mother–placenta–fetus unit operates as a dynamic, multiscale network of physiological subsystems (cardiovascular, autonomic, endocrine, immune, and neural).

Current evidence suggests that the maternal–fetal triad functions as a redundant and cooperative system, supporting maternal brain health through stress reduction and long-term neuroplastic specialization for caregiving. The practical outcomes of this network physiology perspective include the clinical translation of integrated biosignal analysis. By utilizing tools such as the fABAS and monitoring for “network decoupling”—the breakdown of autonomic and molecular synchronizations—clinicians can identify early signatures of fetal distress and developmental vulnerability weeks before the onset of symptomatic pathology (Samjeed et al., 2024b).

Transient, nonlinear couplings—mediated by placental conduction of hemodynamic fluctuations, hormones, cytokines, extracellular vesicles, and neurotransmitters—generate emergent adaptive states that orchestrate fetal neurodevelopment and maternal neuroplasticity. These interactions are bidirectional: maternal network dynamics program fetal brain networks, while fetal/placental signals reshape maternal brain architecture. Disruptions (e.g., in PE or FGR) decouple the network, contributing to the developmental programming of long-term risk, as emphasized in research on autonomic malfunction and delayed neurological maturation (Hoyer et al., 2017).

## How network interactions shape fetal neurodevelopment

Fetal neurodevelopment emerges from synchronized interactions across scales: maternal autonomic/endocrine signals → placental transmission → fetal ANS and brain network formation. The main branches in this scenario are as follows (Sferruzzi-Perri and Camm, 2016; Ivanov et al., 2016; Lakhno, 2017; Hoyer et al., 2017; Hoyer et al., 2019):

- Maternal–fetal HRV coupling as a core network mechanism: Bidirectional cardiac couplings (quantified via partial directed coherence or phase-rectified signal averaging) evolve across gestation, shifting from fetal-to-maternal dominance early to stronger maternal-to-fetal influence later. These transient synchronizations reflect shared ANS regulation and directly index fetal autonomic maturation. Stronger, organized coupling supports progressive vagal dominance, increasing fetal heart rate variability (fetal HRV: SDNN, RMSSD, and STV) and behavioral state coordination—key precursors to brainstem, hypothalamic, and cortical network integration. Weakened coupling (as in preeclampsia/FGR) leads to sympathetic overdrive, reduced fetal HRV, and delayed fABAS, programming altered neurobehavioral trajectories (e.g., poorer cognitive/motor outcomes and temperament dysregulation).
- Placenta as a conductor of hemodynamic and molecular fluctuations: The placenta transmits maternal oscillatory signals (e.g., ∼0.5 Hz RSA-linked peaks in umbilical venous flow velocity spectra) that “mirror” maternal and fetal HRV and stabilize fetal circulation. This conduction supports continuous nutrient/oxygen delivery and modulates fetal ANS tone. The placenta does not merely transfer nutrients; it independently synthesizes and secretes vital neuroactive molecules. Specifically, the placenta produces serotonin (5-HT) from maternal tryptophan to regulate fetal thalamocortical wiring and cell proliferation. Furthermore, the synthesis of the neurosteroid ALLO is critical for fetal myelination and cerebellar development; its reduction due to placental stress is linked to impaired social behavior networks in offspring. Placental-derived factors (serotonin, ALLO, and cytokines) further influence fetal thalamocortical, prefrontal-limbic, and energy-homeostasis circuitry. Maternal stress or inflammation alters these signals, reshaping fetal functional connectivity (e.g., frontoparietal, striatal, and temporoparietal networks observed via fetal MRI).
- Emergent network properties and programming: fetal brain controllability (a network metric of how one region influences others) co-develops with synaptic maturation and synchronizes with maternal brain controllability in a U-shaped inverse trajectory during pregnancy. Higher-order associative networks (e.g., default mode) emerge prenatally, with maternal network dynamics providing the scaffold. This exemplifies network physiology: multiscale couplings (molecular → hemodynamic → autonomic → neural) generate robust fetal connectome topology, while decoupling (e.g., lost 0.5 Hz mirroring in FGR) accelerates sympathetic dominance and programs DOHaD-related vulnerabilities (neurodevelopmental, cardiometabolic, and psychiatric risks).

Present studies in high-risk cohorts operationalize this: preserved maternal–fetal hemodynamic–autonomic coupling supports timely neurological maturation; its disruption in FGR manifests as fetal distress and long-term programming effects.

### How network interactions shape maternal neuroplasticity

Pregnancy triggers one of the most profound periods of adult neuroplasticity, driven by reciprocal fetal–placental–maternal network feedback. These changes reorganize maternal brain networks to support caregiving while integrating fetal signals. Hormonal and placental drivers of structural/functional remodeling: placental hormones (estrogen, progesterone, hCG, and oxytocin) and fetal-derived signals (via EVs and neurotransmitters) induce selective gray matter (GM) reductions—most pronounced in the default mode network (DMN) and frontoparietal networks—alongside white matter expansion and enhanced connectivity in limbic/reward circuits (amygdala, hypothalamus, and nucleus accumbens). This U-shaped trajectory (steepest decline in late pregnancy and partial postpartum recovery) is not loss but specialization: DMN changes enhance theory-of-mind and infant-cue responsiveness, while salience/attention networks heighten maternal vigilance (Kramer et al., 2023). Bidirectional network synchronization: Fetal signals (e.g., via placental conduction of autonomic/hemodynamic fluctuations) feedback to modulate the maternal ANS and HPA axis, promoting neuroplastic windows. Maternal–fetal HRV coupling, for instance, may reflect shared autonomic states that reinforce maternal reward/motivation networks. Longitudinal fMRI shows pregnancy alters resting-state connectivity, particularly DMN coherence, aligning maternal self-referential processing with infant-oriented states. These adaptations persist postpartum, supporting bonding and long-term maternal behavior (DiPietro et al., 2023; Hoyer et al., 2019). Network-level emergent outcomes: The “maternal network” (cortical–limbic–sensory integration) arises from transient couplings across endocrine, immune, and autonomic subsystems. Positive maternal factors (e.g., social support and health behaviors) can buffer stress-related fetal network alterations, illustrating network resilience. Disruptions (e.g., chronic stress) amplify decoupling, with lasting effects on both maternal and fetal trajectories (Ivanov et al., 2016).

### Integrated perspective and implications

Network interactions exemplify multiscale orchestration: horizontal (maternal–fetal subsystem couplings) and vertical (molecular → organ → behavioral) dynamics create adaptive phenotypes. In health, they synchronize fetal ANS/brain network maturation with maternal neuroplastic specialization for caregiving. In pathology, decoupling propagates maladaptive programming. This directly addresses the systemic approach, highlighting biosignal network analysis (HRV, Doppler spectra, and fMRI connectomics) for predictive monitoring and interventions. Clinically, integrated assessment of maternal–fetal couplings offers early biomarkers for neurodevelopmental risk while supporting maternal brain health through stress reduction. The practical outcomes of this review are reframing the mother–placenta–fetus relationship as a unified, adaptive multiscale network, which leads to several concrete diagnostic, therapeutic, and preventive applications. The main value is a transition toward personalized, predictive obstetrics where integrated biosignal analysis and placental monitoring are used to prevent chronic adult diseases through early intrauterine intervention.

1. Advanced diagnostic and monitoring tools:
  - Fetal neurological assessment: Monitoring the synchronization (coupling) of maternal and fetal HRV offers a powerful, non-invasive window into the maturation of the fetal autonomic nervous system (Lakhno, 2025).
  - Predictive biomarkers: Metrics such as the fABAS can quantify neurological maturation and detect developmental vulnerabilities caused by maternal stress or malnutrition weeks before delivery (Hoyer et al., 2017; Hoyer et al., 2019).
  - Decoupling detection: Spectral analysis of umbilical venous blood flow functions as a physiological “mirror” of maternal rhythms; a breakdown in this mirroring serves as an early-warning signal for pathologies such as PE and FGR (Lakhno, 2017).
2. Identifying long-term health risks:
  - DOHaD programming: By analyzing placental signaling (such as mTOR, AMPK, and insulin pathways), clinicians can identify infants at the highest risk for adult diseases, including obesity, Type 2 diabetes, and hypertension (Faa et al., 2024).
  - Neurodevelopmental insights: Changes in placental stress sensors such as OGT provide sex-specific indicators for an offspring’s neurocognitive development and future neuropsychiatric health (Sajdel-Sulkowska, 2023).
3. Emerging therapeutic interventions:
  - Placenta-targeted therapy: The review highlights the potential for localized cargo delivery to the placenta—using viral vectors, nanoparticles, or microbubbles—to improve blood flow and nutrient transport without negatively impacting the mother or fetus directly (Jacobsen et al., 2023).
  - Immunotherapy for pregnancy loss: Novel targets such as PD-1 and Tim-3 have been identified as potential pathways for restoring immune tolerance and treating recurrent miscarriage (Jacobsen et al., 2025).
4. Psychological and public health strategies:
  - Relational support: Because the quality of current relationship care is the strongest predictor of prenatal attachment and acts as a “funnel” for transmitting intergenerational parenting patterns, clinical care should prioritize the emotional health of both parents (Jespersen et al., 2024).
  - Gut–brain axis management: There is a practical need for health guidelines to manage the maternal gut microbiota through diet and stress reduction as these factors cross the placenta to program the offspring’s central nervous system (Barrientos et al., 2024).
  - Postnatal clinical awareness: The persistence of microchimeric cells for decades indicates that a mother’s long-term health (e.g., risk for autoimmune diseases or cancer) is permanently linked to her past pregnancies, requiring more integrated longitudinal care and lifestyle optimization (Jacobsen et al., 2023).

This review reframes the maternal–fetal relationship not as isolated signaling events but as a paradigmatic example of network physiology in action: a unified, adaptive, and multiscale network. It is important to emphasize that, although the individual domains synthesized here—ranging from attachment theory to genomic imprinting—are grounded in the established primary literature, the overarching network physiology framing and the concept of “network decoupling” represent the authors’ own integrative synthetic framework layered onto these disparate fields.

The integrity of this “communicatome” is essential for sustaining a healthy pregnancy. The decoupling of this physiological network—manifesting as weakened HRV synchronization, loss of the umbilical venous spectral “mirror,” or altered placental nutrient signaling—serves as a critical early-warning signature for pregnancy pathologies. Although PE and FGR are primary manifestations of this decoupling, PTB is also a significant consequence, potentially mediated by placental stress and the transfer of inflammatory cargo via placental EVs (Jacobsen et al., 2025; Jacobsen et al., 2023).

The practical outcomes of this perspective include the clinical translation of integrated biosignal analysis. By utilizing tools such as the fABAS and spectral analysis of the umbilical vein—while acknowledging the ductus venosus as a more sensitive marker of early fetal cardiac compromise—clinicians can identify developmental vulnerabilities weeks before the onset of symptomatic pathology. Ultimately, this framework suggests that the maternal–fetal triad functions as a redundant and cooperative system supporting both fetal neurodevelopment and maternal neuroplasticity, specializing the maternal brain for caregiving while reducing stress-related vulnerabilities. Future research must prioritize longitudinal human cohorts to validate the mechanistic insights currently inferred from rodent models, particularly regarding sex-specific stress thresholds and the functional impact of chimeric cells.

In summary, although the integration of physiological, molecular, and cellular signals defines the maternal–fetal triad, many of the specific “coding” mechanisms remain to be fully established in humans. For example, the loss of fetal and maternal hemodynamic coupling could be considered a presumable pathogenetic mechanism of fetal distress in PE. Similarly, although the metabolic and stress-sensing pathways identified in rodent studies provide high-resolution mechanistic detail, they should be viewed by clinicians as plausible biological frameworks that require further validation through rigorous human longitudinal cohorts. By distinguishing between established human clinical markers and mechanistic insights inferred from rodent models, this review aims to provide a balanced perspective on the current state of DOHaD research. By reframing pregnancy through network physiology, we identify that the maternal–fetal triad functions as a redundant, cooperative system. The practical outcomes of this framework include the clinical use of the fABAS and the monitoring of network signatures to detect developmental vulnerabilities weeks before the onset of symptoms. This transition toward personalized, predictive obstetrics enables early intrauterine intervention to prevent chronic adult diseases.

Limitations: As a narrative synthesis within the framework of network physiology, this review is subject to several limitations. First, there is uneven domain coverage across the “communicatome.” Although the physiological and autonomic coupling sections are grounded in a substantial body of clinical data (including HRV metrics and Doppler spectral analysis), the genetic domain discussion is comparatively thin, relying on a limited number of sources focused on imprinting and conflict theory. Furthermore, the small total sample of included papers (n = 38) reflects a targeted scoping approach rather than an exhaustive systematic review, which may introduce selection bias. Consequently, the findings presented here should be interpreted as an integrative synthetic framework rather than a comprehensive or definitive census of all maternal–fetal interactions.

## Conclusion

The emerging field of network physiology provides a transformative framework for understanding human development, reframing the relationship between the mother and fetus from two separate entities into a unified, adaptive, and multiscale triad consisting of the mother, placenta, and fetus. This triad is coupled through complex, bidirectional feedback loops—ranging from molecular epigenetic modifications to systemic autonomic synchronizations—that orchestrate healthy gestation and program long-term health.

Central to this physiological cross-talk is the coupling of maternal and fetal HRV, which serves as a non-invasive window into the maturation of the fetal ANS. Research highlights that this synchronization is dynamic, characterized by a shift in directionality shift from fetal-to-maternal influence in early pregnancy to a dominant maternal-to-fetal drive by mid- to late gestation. The umbilical vein acts as a physiological “mirror” of maternal autonomic rhythms, conducting oscillations such as RSA across the placental barrier to stabilize fetal circulation and modulate neurodevelopment.

This integration extends beyond biosignals into a complex biological legacy defined by cellular and extravesicular chimerism. Fetal and maternal cells and EVs are exchanged and persist in host tissues for decades, modulating immune tolerance, aiding tissue repair, and influencing the risk of autoimmune or cancerous diseases. The placenta acts as a sophisticated nutrient sensor (via mTOR and OGT signaling) that prioritizes maternal supply signals over fetal demand to ensure mutual survival.

Simultaneously, the maternal gut microbiota produces metabolites such as SCFAs that cross the placenta to program the development of the fetal gut–brain axis. Maternal–fetal and paternal–fetal attachment are not only predicted by physical cues such as fetal movement but are also part of a transgenerational process in which the quality of care in the current parental relationship serves as the primary channel for transmitting past childhood experiences to the next generation.

The decoupling of this network—manifesting as weakened HRV synchronization, loss of the umbilical venous spectral “mirror,” or altered placental nutrient signaling—serves as an early warning for pathologies such as PE and FGR. Clinically, tools such as the fABAS offer the potential to monitor neurological maturation and detect vulnerabilities caused by maternal stress or malnutrition weeks before birth.

By reframing pregnancy through network physiology, we identify that the maternal–fetal triad functions as a redundant, cooperative system. The practical outcomes include the clinical use of the fetal fABAS and the monitoring of network signatures—such as ductus venosus indices and EV cargo—to detect developmental vulnerabilities weeks before the onset of symptoms. This transition toward personalized, predictive obstetrics enables early intrauterine intervention to mitigate the lifelong risk of adult chronic disease.

In summary, the mother and fetus function as an adaptive multiscale network where physiological integrity is essential for successful pregnancy outcomes. The shift toward personalized surveillance demands the clinical integration of multiscale biosignals. By understanding the mother–placenta–fetus triad as a synergetic model of coupled oscillators and molecular networks, clinicians can better predict and mitigate neurodevelopmental risks and the long-term sequelae of developmental programming. Future research must prioritize longitudinal, multi-generational studies and the development of standardized protocols for integrated biosignal analysis to fully operationalize these insights for personalized obstetric care and the prevention of chronic adult diseases through early intrauterine intervention.

## Glossary

### General concepts and frameworks

ANS: Autonomic nervous system
BMI: Body mass index
DOHaD: Developmental Origins of Health and Disease (the “Barker Hypothesis”)
GBA: Gut–brain axis
MFA: Maternal–fetal attachment
MFAS: Maternal fetal attachment scale

### Physiological and network analysis

AMo: Amplitude of mode
BPRSA: Bivariate phase-rectified signal averaging (analytical method for heart rate coupling)
fABAS: Fetal Autonomic Brain Age Score
fHRV/HRV: Fetal heart rate variability/heart rate variability
HRJSD: High-resolution joint symbolic dynamics
mHR: Maternal heart rate
PDC: Partial directed coherence (analytical method for heart rate coupling)
RMSSDs: Root mean square of successive differences (HRV metric for vagal activity)
RSA: Respiratory sinus arrhythmia
SDNN: Standard deviation of normal-to-normal intervals (overall HRV metric)
SI: Stress index
STV: Short-term variation

### Molecular biology, metabolism, and chimerism

ALLO: Allopregnanolone (placental-derived neurosteroid)
EVs: Extracellular vesicles
FMc: Fetal microchimeric cells
GR: Glucocorticoid receptor
hCG: Human chorionic gonadotropin
HPA: Hypothalamic–pituitary–adrenal axis
IGF-2/IGF-II: Insulin-like growth factor-2
IGF-2R: IGF-2 receptor
MMc: Maternal microchimeric cells
mTOR: Mechanistic target of rapamycin (cellular nutrient sensor)
OGT: O-GlcNAc transferase (placental stress/nutrient sensor)
PlGF: Placental growth factor
SCFAs: Short-chain fatty acids
sFLT1: Soluble fms-like tyrosine kinase 1 (anti-angiogenic factor)

### Immunology and clinical outcomes

dNK: Decidual natural killer cells
FGR/IUGR: Fetal growth restriction/intrauterine growth restriction
M2: Alternatively activated (anti-inflammatory) macrophage phenotype
PE: Pre-eclampsia
Tregs: Regulatory T cells

### Neuroimaging and brain structure

DMN: Default mode network
fMRI: Fetal magnetic resonance imaging
GM: Gray matter
